# Clonidine as an Adjunct for the Successful Treatment of Labor Epidural Infusion-Associated Interscapular Pain

**DOI:** 10.7759/cureus.41179

**Published:** 2023-06-30

**Authors:** Alexander G Samworth, Alexander M DeLeon, Samir Patel, Emery McCrory, Carmen Lopez, Jason Farrer

**Affiliations:** 1 Anesthesiology, Northwestern University Feinberg School of Medicine, Chicago, USA

**Keywords:** cesarean delivery, fentanyl, interscapular pain, epidural, clonidine

## Abstract

Epidural infusion-associated interscapular pain for laboring parturients is a treatment dilemma for obstetric anesthesiologists. We report a case of a parturient who was successfully treated for labor epidural analgesia-associated interscapular pain. Our treatment plan included reducing the volume of local anesthetic administered by adding the adjunct of clonidine, increasing the epidural solution concentration of local anesthetic, and reducing the overall infusion rate. We conclude that epidural clonidine should be considered a safe adjunct when treating laboring parturients who report epidural infusion-associated interscapular pain.

## Introduction

Balancing the side effects of epidural labor analgesia infusions with the beneficial effects can be a clinical challenge for obstetric anesthesiologists. The Comparative Obstetric Mobile Epidural Technique Study Group (COMET) reported the results of a randomized controlled trial evaluating the effects of a concentrated bupivacaine (0.25% bupivacaine) solution compared to a dilute solution (0.1% bupivacaine with 2 mcg/mL of fentanyl) for labor analgesia [1]. The COMET study group showed a 23% reduction in the incidence of operative vaginal delivery (e.g., forceps delivery) when dilute solutions are used compared to concentrated labor epidural analgesia solutions. A reported consequence of dilute solutions is an increase in the volume of solution in a parturient's epidural space, which may lead to pressure-induced interscapular pain associated with bolus dosing of an epidural [2,3]. The use of clonidine as part of a treatment protocol has not been previously reported for treating labor epidural-analgesia-associated interscapular pain.

We report the first case of labor epidural analgesia-associated interscapular pain successfully treated with epidurally administered clonidine in addition to alterations in the epidural infusion concentration and rate.

## Case presentation

A 35-year-old, spontaneously laboring G1P0 parturient at 39.3 weeks (BMI of 39.1 kg/m^2^) and no significant past medical history presented for epidural labor analgesia at 3 cm dilation. An uncomplicated combined spinal epidural (CSE) was initiated with 15 mcg of fentanyl and two milligrams of bupivacaine in the intrathecal space at the L3-4 level with a single attempt and no complications with placement. A programmed intermitted epidural bolus (PIEB) infusion of 0.0625% bupivacaine and fentanyl, 2 mcg/mL, was initiated for continuous labor analgesia. The institutional protocol consisted of a programmed bolus of 10 mL of the epidural solution every 60 minutes. A patient-controlled epidural analgesia bolus (PCEA) of 8 mL every 10 minutes was programmed for breakthrough pain. The infusion was set for an hourly limit of 32 mL of the epidural solution.

Eight hours after initiating the infusion, the patient complained of "throbbing" pain between her shoulder blades with each PIEB or PCEA dose. She rated the pain worse than contraction pain, scoring the pain as eight out of 10 on the visual analog pain scale (VAS). She considered discontinuing her epidural infusion if the interscapular pain could not be resolved.

Upon investigation, it was noted that she had received a cumulative epidural volume of 176 mL over eight hours. The patient's sensory level was T8 bilaterally to cold, and there was no evidence of catheter dislodgement. The patient did not report labor contraction pain; her cervical dilation was 3.5 cm. To address her interscapular pain, she received 75 mcg of epidural clonidine. Additionally, given that the presumed etiology of her complaint was excessive epidural fluid volume, the epidural solution was switched to 0.1% bupivacaine with no change in fentanyl concentration. The PIEB was discontinued, and a continuous infusion was started at 8 mL every hour, with the PCEA dose reduced to 6 mL per hour.

The patient complained of no further interscapular pain within 30 minutes and did not receive additional "top-up" doses of bupivacaine for the remainder of her labor. She underwent a successful cesarean delivery four hours later for the indication of the arrest at six centimeters dilation. Her epidural was dosed for surgical anesthesia with 15 mL of 2% lidocaine containing bicarbonate at a 1 to 10 dilution, and epinephrine at 5 mcg/mL. Her surgical epidural dose also included 100 mcg of fentanyl. The cesarean delivery proceeded without incident.

The remainder of the hospital course was uneventful, and the patient was discharged on postpartum day two.

## Discussion

Klumpner et al. published a case series describing labor epidural analgesia-associated interscapular pain [3]. Their group reported several patients who received epidural analgesia for up to 19 hours and experienced intense interscapular pain associated with bolus doses. The treatments described by the authors included increasing the epidural concentration of local anesthetic to reduce the volume delivered, administration of epidural fentanyl, and even replacement of the epidural catheter [3]. The use of epidural clonidine was not described in their report.

Clonidine has been a safe and effective addition to the tools for anesthesiologists caring for laboring patients [4-7]. The dose of clonidine (75 mcg) used in the case was consistent with Landau et al. [4]. It has been reported to have dose-sparing effects when added to ropivacaine with minimal effects on both the mother and neonate [4,7]. The rationale for the use of clonidine, which is an alpha-2 agonist, for the treatment of epidural-associated interscapular pain is two-fold [8]. First, clonidine has been specifically studied and shown to be effective when administered epidurally for spine surgery, indicating efficacy in treating the pain associated with the vertebral column [8]. Second, the dose-sparing effects of clonidine would likely reduce the need for patient-controlled local anesthetic boluses to treat labor contraction-associated pain [4,5].

The dilute epidural solution used in the COMET study was the same one used in the presented case after the epidural clonidine was administered [1]. Our patient underwent cesarean delivery, so the possibility of our change in bupivacaine solution or clonidine administration contributing to the need for cesarean delivery is valid. Yet, the findings of the COMET study were that there was no association between an increased rate of cesarean delivery with more concentrated epidural infusion solutions [1]. Similarly, epidural clonidine has not been associated with an increased cesarean delivery rate [4,9].

**Figure 1:**
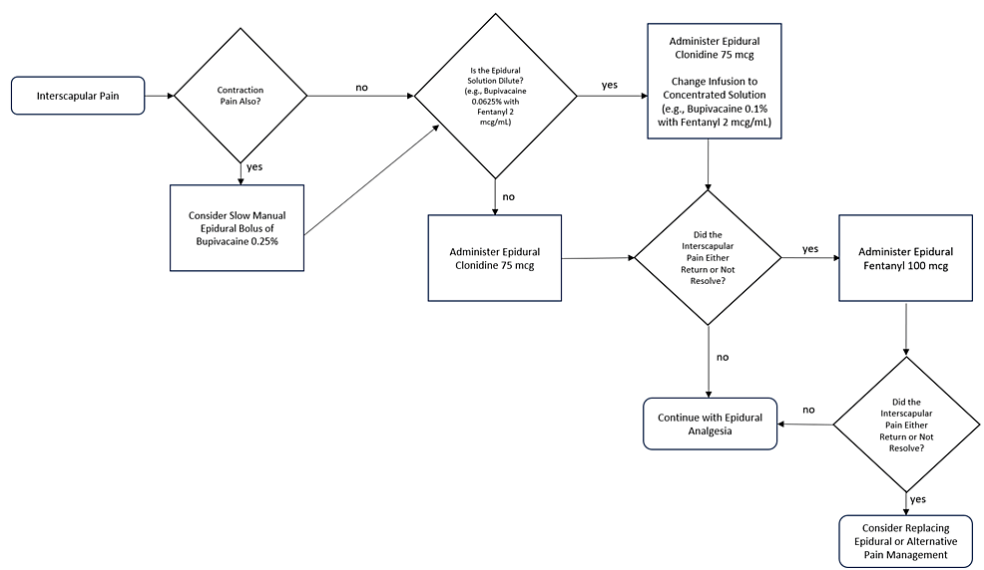
A Suggested Protocol for Epidural-Associated Interscapular Pain The authors suggest the illustrated protocol when dealing with a patient with labor epidural-associated interscapular pain during labor analgesia.

The side effects of other epidural modalities, such as fentanyl, include nausea, pruritis, and sedation [7]. Despite concerns for potential hypotension with epidurally administered clonidine, it is not associated with clinically significant drops in maternal blood pressure [7,9]. Clonidine is also not associated with nausea, pruritis, or sedation to the degree of epidural fentanyl and is safe for both the neonate and mother in labor [7,9]. A suggested protocol is illustrated below (Figure 1).

The limitation of this report is its generalizability, given that it is based on a single patient. This case study is intended to be a backbone for future research into the mechanism, incidence, and treatment of labor epidural infusion-related interscapular pain. Multiple interventions were made, yet the therapy's addition of clonidine had not been previously reported.

## Conclusions

Dilute epidural infusions of local anesthetic with opioids are commonly used for labor analgesia. Clinicians treating patients who experience labor epidural interscapular pain may desire alternatives to epidural opioids due to opioid-associated side effects or if epidural opioids are ineffective in a given patient. Epidural clonidine administration should be considered when managing a laboring patient reporting epidural bolus-associated interscapular pain.

## References

[1] Shennan AH (2001). Effect of low-dose mobile versus traditional epidural techniques on mode of delivery: a randomised controlled trial. Lancet.

[2] Dickerson DM, Dai R, Scavone BM, McDade W (2016). Labor epidural intolerance due to a congenitally narrowed spinal canal. Reg Anesth Pain Med.

[3] Klumpner TT, Toledo P, Wong CA, Farrer JR (2016). Interscapular pain associated with neuraxial labour analgesia: a case series. Can J Anaesth.

[4] Landau R, Schiffer E, Morales M, Savoldelli G, Kern C (2002). The dose-sparing effect of clonidine added to ropivacaine for labor epidural analgesia. Anesth Analg.

[5] Aveline C, El Metaoua S, Masmoudi A, Boelle PY, Bonnet F (2002). The effect of clonidine on the minimum local analgesic concentration of epidural ropivacaine during labor. Anesth Analg.

[6] Fernandes HS, Bliacheriene F, Vago TM, Corregliano GT, Torres ML, Francisco RP, Ashmawi HA (2018). Clonidine effect on pain after cesarean delivery: a randomized controlled trial of different routes of administration. Anesth Analg.

[7] Cigarini I, Kaba A, Bonnet F, Brohon E, Dutz F, Damas F, Hans P (1995). Epidural clonidine combined with bupivacaine for analgesia in labor. Effects on mother and neonate. Reg Anesth.

[8] Farmery AD, Wilson-Macdonald J (2009). The analgesic effect of epidural clonidine after spinal surgery: a randomized placebo-controlled trial. Anesth Analg.

[9] Parker RK, Connelly NR, Lucas T, Serban S, Pristas R, Berman E, Gibson C (2007). Epidural clonidine added to a bupivacaine infusion increases analgesic duration in labor without adverse maternal or fetal effects. J Anesth.

